# Observations on the Management of Enteric Fever, Etc.

**Published:** 1883-05-20

**Authors:** James C. Wilson

**Affiliations:** Physician to the Jefferson Medical Hospital, and to the Philadelphia Hospital


### OBSERVATIONS ON THE MANAGEMENT OF ENTERIC FEVER ACCORDING TO A PLAN BASED UPON THE SO-CALLED SPECIFIC TREATMENT.

By JAMES C. WILSON, M. D.,
Physician to the Jefferson Medical Hospital, and to the Philadelphia Hospital.
(Read January 3, 1883.)

I desire to lay before the college a plan of managing enteric fever, which I have employed during the past year, and which, tested by such uncertain but not necessarily fallacious means as are available for a limited series of cases, has yielded satisfactory results.

The object of this communication will, I believe, be best attained by first sketching in outline the plan of treatment itself; next, by reviewing the considerations which led to its adoption, and finally by a brief study of the cases. This arrangement of the topics will enable us to economize time.

*The Plan of Treatment.*—The scope of this paper, and the necessity to be brief, debar me from the consideration of the general management of the patient, dietetics, the treatment of complications and sequels and of the prophylaxis; and restrict me, in the main, to the subject of the management by medicinal means. It is, in fact, this part of the treatment that, superadded to so-called rational and expectant method in general use in this community, differs from the common practice and constitutes the plan in question.

So soon as the patient is found to have enteric fever. or, in many instances, so soon as his symptoms warrant a reasonable suspicion that he is about to develop it, he is put to bed, ordered a diet consisting of milk, animal broths, jelly and simple custards, in small amounts, and at intervals of two or three hours. At night he is given a dose of calomel. This dose varies in amount from 7½ to 10 grains (0.5 to 0.66 gramme) and is repeated every second evening until three or rarely four doses have been administered in the course of the first six or eight days. It is given alone or in connection with sodium bicarbonate. There is commonly a slight increase of diarrhœa, if it be present, without aggravation of the other symptoms, and in some instances the tendency of the temperature at this time to steadily rise, appears to be controlled. If, as is frequently the case, spontaneous diarrhœa has not recurred in the first week, the calomel usually brings about two large evacuations on the day following its administration, not more. In either case the tendency to frequent passages in the later stages of the attack is favorably influenced by the repeated administration of this drug during the first week. If the case does not come under observation until the 10th day, one. or at most, two doses of calomel are given. No further doses of it are, however, given during the course of the attack unless constipation occurs. In this event if the evidences of extensive or deep implication of the intestinal wall, such as abdominal pain, tenderness or marked tympany are absent, calomel in $7\frac{1}{2}$ grain (0.5 gramme) doses is given at intervals of three or four days. If there is reason to suspect serious intestinal lesions, the lower bowel may be more safely emptied of its contents every third or fourth day, by enemata of moderate size (8 to 10 fluid ounces). It is necessary to bear in mind that the gravest lesion of the gut leading even to hemorrhage and perforation, have occasionally been observed in cases characterized, not only by constipation, but also by an entire absence of pain or tenderness, and very moderate tympany. The danger of salivation from calomel in these doses in enteric fever appears to be slight. In only one case in sixteen were the mercurial fetor and slight swelling of the gums observed.

Excessive diarrhœa has been controlled by the use of opium, either in suppositories, containing 1 grain (0.06 gramme), or by the mouth in quarter-grain (0.016 gramme) doses, often associated with bismuth, and given *pro re nata.* It is an invariable rule that the patient be kept in the horizontal position and to the use of the bed-pan and urinal, from the time of the recognition of the disease until defervescences is completed. He is, however, turned upon his side from time to time, and made to maintain that position for twenty or thirty minutes, if necessary, being supported by the nurse.

From the beginning of the attack the following mixture is regularly administered in doses of one, two or even three drops in a sherry-glassful of ice-water after food, every two or three hours during the day and night:

| | | | |
|---|---|---|---|
| ℞ Tinct. iodinii, | f ʒ ij | ................8 | 00 c. c. |
| Acid. carbolici liq., | f ʒ j | ..................4 | 00 c. c. |

M. Unless some unusual circumstance occur to render a change necessary, this medicine is not suspended until the attack draws to a close. It is well borne by the stomach and excites no repugnance on the part of patients. In one case only has it been necessary to omit the carbolic acid on account of the disgust assumed by its odor.

Partly for the sake of its favorable influence upon the skin and for the sake of cleanliness; partly because of its favorable though slight influence upon the temperature, the patient is to be sponged twice a day with equal parts of aromatic vinegar or alcohol, and cold water. If it is more grateful to him, the sponging may be done in tepid water, the evaporation of an extensive film of water not below the temperature of his body probably being not without a refrigerating tendency.

When the evening axillary temperature reaches 104° F. (40° C.) quinine in massive doses, 24 to 30 grains (1.66 to 2.00 grammes) is given upon a falling temperature. I usually direct 8 to 10 grains to be given in solution at 5, at 5:30, and at 6 a. m. the following morning. Administered thus at the decline of the temperature in its diurnal revolution, these large doses of quinine depress it from 2.5° to 3.5° F. (1.4° to 1.8° C.). After the lapse of forty-eight to seventy-two hours, it necessary the dose may be repeated. If these doses be rejected by the stomach—an unusual circumstance —half the quantity of quinine may be administered hypodermically. For this purpose a citric acid solution is to be preferred. Since the adoption of the plan of treatment under consideration, I have not encountered cases attended with such hyperpyrexia as has rendered attempts to control it by cold baths necessary or even advisable.

The minor nervous symptoms are best held in check by skillful nursing. For the relief of the headache of the first ten days, absolute quietude, a dim light, etc., are often sufficient; occasionally the bromides alone, or in combination with chloral, are required. Later in the course of the disease chloral is unsafe. From the end of the first week the patient cannot be left unattended, even for a few minutes, without risk. Persons in whom delirium was only occasional and transient, have in many instances destroyed themselves during the momentary absence of the nurse.

Alcohol is not often indicated prior to the beginning of the third week. It may, however, by reason of the habits of certain patients, be necessary throughout the attack. Although forming no essential part of the treatment, it is commonly administered in varying though usually small amounts towards the close of the sickness. Some patients do well without taking it at all. It is of course administered in accordance with well-understood indications upon the supervention of delirium, ataxic symptoms and the evidences of failures of the forces of the circulation. The patients are carefully watched well into convaleseence, and cautioned against too soon regarding themselves restored to health.

The dangers of the establishment of a focus of contagion are guarded against by the systematic, thorough disinfection of the stools immediately after they are voided.

The considerations which induced me to adopt this plan of treatment indicated in the foregoing sketch, are :

1. A feeling of dissatisfaction regarding the expectant method of treating enteric fever. This feeling, vague at first, grew more definite and stronger with clinical opportunities, and a fuller knowledge of the natural history of the disease, until it became a motive, impelling me to cast about for some different and more satisfactory plan. This feeling has been during the past decade a very general one in the profession in all parts of the world, as is attested by an almost endless succession of journal articles setting forth new plans of treatment, and the use of new drugs in the management of this, the most common and most important of the acute infectious diseases of the present epoch in medical history. Most of the plans thus suggested have led to disappointment when tested by the fuller observations of the profession ; many of them have failed to attract general attention, and some few are still *sub judice.* Their number and diversity bear witness to a wide-spread distrust of the once well-established expectant treatment.. This distrust is, however, based upon something more tangible than a mere feeling of dissatisfaction. The statistics of all observers whose cases have been sufficiently numerous to be trustworthy, show enteric fever to be, when treated by the expectant plan, a disease of high death rate.

2. Enteric fever is the very type of the general diseases, of affections *totius substantiæ.* The tissues are universally implicated in the morbid processes; no function of the body wholly escapes perturbation. For this reason, plans of treatment suggested by the prominence of certain groups of symptoms, or by the known lesions of particular organs, even though of undoubted benefit as far as they go, are in theory unsatisfactory, because they are directed in effect against conspicuous manifestations of the cause of the sickness rather than against the cause itself

Whilst in actual practice, the treatment by turpentine, by alcohol, by opium with lead, or the silver nitrate, or by agents capable of controlling the febrile movements, as quinine, digatalis, salicin, and the salicylates, even the cold-water treatment itself, although at times, and in the hands of certain clinicians showing favorable results—all these have failed of general acceptance on the part of the profession.

3. The general character of. the disease, the specific nature of its cause, the unsatisfactory results alike of an expectant and of a symptomatic plan of treatment, or rather of the two combined, have united to render the idea of a specific treatment, a true cure for enteric fever, a most attractive one, to stimulate thoughtful observers to renew again and again the disappointing search for it. To this idea may be traced the treatment by the mineral acids, by quinine alone, by quinine and digitalis, by iodine, by the potassium iodide, by calomel.

4. Not only is the conception of a specific treatment for specific diseases a most attractive one, and the attainment of such a treatment for enteric fever brought within the bounds of a reasonable hope by the analogy of syphilis and the malarial diseases, but the search after it with due caution and judgment, has also the warrant of the very highest medical authority.

The treatment adopted is thus seen to consist of the use of the two remedies that are proved to exert a favorable influence upon the disease, iodine and calomel, with the addition of carbolic acid in minute amounts. I am aware that no postive conclusions as to the efficacy of particular plans of treatment can be deduced from a limited series of cases. I am also aware that few acute diseases show greater variations in intensity and in the percentage of mortality at different periods, and under different circumstances, than enteric fever. Nevertheless, I have ventured to occupy your attention with this subject to-night, because the results of the treatment encourage me to hope that its discussion in this way will lead to its trial on a more extended scale. That it amounts to a specific treatment in the narrow sense is not affirmed. It is tentative, provisional, but it is, nevertheless, to be regarded as a contribution to the subject of the specific treatment of enteric fever.

The total number of cases treated by this plan is sixteen; all recovered, one being now in the second week of convalescence.

Of these, eight were severe, the temperature reaching or exceeding 104° F. (40° C.).

Of these eight severe cases, one was characterized by uncontrollable vomiting in the third week. The patient retained no food taken by the mouth for five consecutive days.

One case was very irregular in its course, and was complicated by an abdominal abscess which discharged by the bowel. The temperature in this case on two occasions attained 105° F. (40.5°C.) This case presented the characteristic eruption of enteric fever.

A third case was prolonged by a severe relapse.

Of the eight cases in which the observed temperature did not at any time attain 104° F. (40° C.), and which was therefore looked upon as medium or mild cases, one was complicated by crural phlebitis, and another by the occurrence of intestinal hemorrhage.

The average duration of the eight severe cases was about 31 days; that of the eight mild and medium cases was about 25 days.

Of the whole number, ten were treated in hospital, six in private practice. All from the time of their coming under observation were under my personal care.

In two cases the special plan of treatment was abandoned about the beginning of the third week on account of the supervention of unusual symptoms of great gravity. These related respectively to gastric irritability and an obscure abdominal abscess.—*Chicago Med. Jour. & Examiner.*

